# Two cases of cholera O1 in South Batinah, Oman, April 2019: lessons learned

**DOI:** 10.4178/epih.e2019033

**Published:** 2019-07-12

**Authors:** Zayid K. Al Mayahi, Nasser Al-Shaqsi, Hamid A. Elmutashi, Ali Al-Dhoyani, Azza Al Hattali, Khalid Salim, Issa S. Al Fulaiti, Mahmood S. Al Subhi

**Affiliations:** 1 Disease Surveillance and Control Department, Ministry of Health, South Batinah Governorate, Rustaq, Oman; 2 Al Rustaq Hospital, Ministry of Health, Rustaq, Oman; 3 Barka Extended Health Center, Ministry of Health, Barka, Oman

**Keywords:** Cholera, Surveillance, Epidemiology, Stool antigen test, Infection control, Oman

## Abstract

Cholera represents an ongoing threat to many low-income and middle-income countries, but some cases of cholera even occur in high-income countries. Therefore, to prevent or combat cholera outbreaks, it is necessary to maintain the capacity to rapidly detect cholera cases, implement infection control measures, and improve general hygiene in terms of the environment, water, and food. The 2 cases, 1 imported and 1 secondary, described herein are broadly indicative of areas that require improvement. These cases were missed at the primary health care stage, which should be the first detection point even for unusual diseases such as cholera, and the absence of strict infection control practices at the primary care level is believed to contribute to secondary cases of infection. This report also encourages countries to ensure that rapid diagnostic stool tests are available to enable quick detection, as well as to provide information to people travelling to areas where cholera is endemic.

## INTRODUCTION

Many people think that cholera is not a disease that is a modern priority, and that it is a disease from the past; in fact, though, cholera continues to kill tens of thousands of people across the world, including in countries that had never had the disease previously. At least 47 countries are still affected by cholera globally, with about 2.9 million cases and 95,000 deaths per year worldwide [1]. This disease is also a stark marker of inequity, and the countries threatened by it are usually the poorest, with populations that are vulnerable due to a lack of basic necessities such as clean water, safe food, and well-established sanitary infrastructure. Epidemics are also fuelled by natural disasters, such as in Haiti where at least 800,000 cases have been reported, resulting in over 10,000 deaths since the 2010 outbreak [2]. Yemen’s cholera outbreaks were powered by the internal and external conflicts and wars there, and Yemen’s recent outbreak in 2016 caused 2,767 deaths from 4,510 laboratory-confirmed cases, with 1,423,700 suspected cases through January 2019 [3].

Oman, the eastern neighbour of Yemen, is free of endemic cholera; however, imported cases do occur. During the period 1999 to 2015, a total of 40 typical cholera cases were confirmed in Oman, of which the majority (29 cases) were classified as imported (including 2 deaths), mainly from the Indian subcontinent; the remaining 9 cases were secondary to 3 imported cases. During this period, 2 cases were also diagnosed in an Omani family from South Batinah in the year 2003; however, the ultimate source of infection was not identified [4].

This report focuses on an imported case of cholera from Pakistan that resulted in an additional secondary case within the same family. Both cases demonstrate classical scenarios for cholera and highlight the importance of screening contacts, along with the role of rapid diagnostic testing of stool. They also reflect the pressing need to improve public health surveillance, infection control practices, and awareness about such diseases in the community.

## CASE REPORTS

### Case 1: father

A 58-year-old Omani man with known hypertension controlled with lisinopril, from Barka, South Batinah governorate, presented to a nearby polyclinic in the early morning of April 24, 2019, with sudden-onset severe abdominal pain, vomiting, and severe copious watery, non-bloody diarrhoea that had lasted for 12 hours. The patient was sick-looking and severely dehydrated, with reduced skin turgor and Kussmaul breathing. Oxygen saturation was 85% in room air, and his blood pressure was 85/65 mmHg, with a body temperature of 37°C. The patient was started on intravenous fluid through 2 inserted lines. Laboratory investigations were performed, with the following results: haemoglobin, 20.41 g/dL; white blood cells, 13.81 k/μL; creatinine, 396 μmol/L; urea, 6.3 mmol/L; sodium, 142 mmol/L; potassium, 3.47 mmol/L; and estimated glomerular filtration rate, 14.4 μmol/L. He received a total of 1,500 mL of normal saline before being escorted to the regional secondary hospital for further evaluation and management.

The initial evaluation at the secondary hospital immediately raised a suspicion of cholera based on the severe and persistent gastroenteritis and a more detailed history taken from the patient, revealing that he had arrived from Pakistan in the previous afternoon. An electronic notification of suspected cholera was immediately sent to the appropriate authorities, and the regional public health team became involved in further investigation of the case. The patient was also placed under strict enteric precautions. The stool examination showed a watery-rice consistency, and the rapid diagnostic test (RDT) (Crystal VC, immunochromatographic One Step Rapid Test for *Vibrio cholerae*; Dipstick, Arkray Health Care Private, Surat, India) was positive. The stool culture result, which was released on April 28, showed growth of *V. cholerae* O1 E1 Tor, subtype Inaba, which was fully sensitive to ampicillin, chloramphenicol, tetracycline, and trimethoprim/sulfamethoxazole. The patient had already received a dose of doxycycline (300 mg, stat) on April 25, and ultrasonography of the kidney, ureter, and bladder had been performed with normal results.

As the patient’s condition worsened, he was moved to the intensive care unit and started on haemodialysis in order to manage his acute kidney injury. Two days after admission, the patient’s condition had improved thanks to extensive rehydration therapy and haemodialysis. The vomiting, abdominal pains, and diarrhoea ceased slowly, and his good recovery led him to be discharged on May 6. Follow-up appointments and blood investigations revealed improved renal function.

### Field visit

An investigation team consisting of an epidemiologist, infection control staff, and a health inspector contacted the patient’s family and arranged for an urgent home visit on April 26. The family was counselled about the suspicions of cholera in the father and the team obtained further details.

The patient and his wife had been on a 2-week trip to Karachi in Pakistan. Throughout their stay, they had no medical illnesses, and no sick people had stayed at their lodgings or been among their friends and relatives. They had cooked food in their house and had not eaten fast food from street restaurants. They had also bought bottled water for drinking, although they used the local water supply for cooking and showering. They both commented that the local water had not appeared safe to drink, and that it tasted salty. The patient and his wife had arrived in Oman in the afternoon of April 23 in good condition. The patient then started to complain of gastro-intestinal symptoms later that night, about 8 hours after arrival. The patient had multiple episodes of nausea, vomiting, and loose bowel movements, and his wife had to clean up the resulting mess. The patient was then taken to the polyclinic by his daughter and son, who accompanied him at the polyclinic before he was escorted to the secondary hospital. Importantly, the daughter helped with cleaning her father by removing vomitus and secretions directly from his mouth and body. All family contacts (12 members) were thus requested to provide stool samples for screening for cholera by RDT, and 10 samples were received. All contacts were placed under symptom monitoring. Advice was provided to the family, particularly with regard to hand washing, and using disinfectant cleaners to clean the house.

### Case 2: daughter

**Figure 1.**
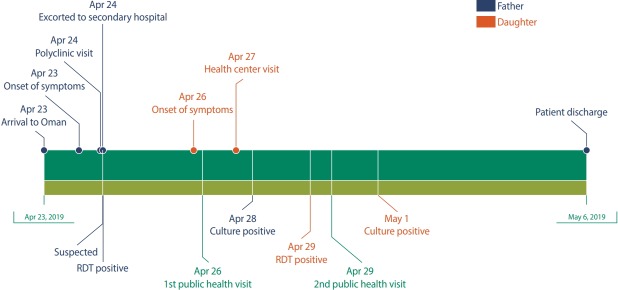
Timeline of patients' clinical progress, tests performed and management. RDT, rapid diagnostic test.

The team was informed that the rapid diagnostic stool test for the patient’s daughter, who was 31 years old, was positive. As every family member denied suffering any symptoms on the first field visit, another visit was conducted to further investigate the possibility of a secondary case in the family. On interviewing the patient at her home, although she looked well and was not dehydrated, she confirmed that she had begun to experience frequent loose bowel movements and moderate to severe abdominal pain on April 26, and that she had sought medical consultation at a nearby health center, where she was given supportive treatment and discharged. When asked why she had not reported this to the public health team, she answered that she thought her symptoms were irrelevant to her father’s condition. The patient was then given a 300 mg stat dose of doxycycline along with hyoscine butyl bromide. She was advised to use a designated toilet, and the need for strict hand washing was emphasised. In 2 more days, the patient’s condition had improved, and her symptoms had disappeared. Her culture report was released on May 1; this also showed growth of a similar isolate of her father, *V. cholerae* O1 E1 Tor, subtype Inaba, fully sensitive to ampicillin, chloramphenicol, tetracycline, and trimethoprim/sulfamethoxazole. Figure 1 presents an overview of the 2 patients’ clinical progress, the tests performed, and their management.

### Diagnostic role of microbiology

The initial workup of the stool samples in the regional hospital laboratory revealed a Gram-negative non-lactose-fermenter coccobacilli curved shape. The stool samples analysed in the regional hospital laboratory were positive on the rapid cholera antigen test. Stool samples were cultured and the growing isolates were sent to the Central Public Health Laboratory (CPHL) on MacConkey agar for identification and confirmation, susceptibility testing, and serotyping. At the CPHL, the isolates were sub-cultured on blood agar and thiosulfate–citrate–bile-salts–sucrose agar and incubated in air at 35-37°C for 18-24 hours. Fermentation of sugars such as glucose, maltose, mannitol, and sucrose tested positive using the Vitek system (Vitek-2 Compact; bioMérieux, Marcy-l'Étoile, France). Most strains were also motile at 37°C, metabolized lysine and ornithine, and showed a positive string test. The isolates were identified using a matrix-assisted laser desorption/ionization biotyper (Bruker, Billerica, MA, USA), which indicated that the bacteria were a *Vibrio* species. Vitek was used for identification and it gave a 98% probability that the isolates were *V. cholerae*.

The agglutination test (*V. cholerae* antisera) used was positive for *V. cholerae* serogroup O1 serovar Inaba. Since the growth showed haemolysis of colonies, the strain was most probably El Tor *V. cholerae*. Antibiotic susceptibility testing was done using disc diffusion for ampicillin, tetracycline, chloramphenicol, and trimethoprim/sulfamethoxazole according to the relevant guidelines (M100, 29th edition, Clinical and Laboratory Standards Institute).

### Ethics statement

Written informed consent was obtained from the patient for publication of this case report and any accompanying images.

## DISCUSSION

It is not uncommon to see imported cases of cholera in Oman for many reasons. A large proportion of Oman’s expatriates and labour force come from the Indian subcontinent and multiple African countries, and these areas of the world, along with the Caribbean, are currently the most endemic with cholera [5]. Many of these outbreaks can be attributed to consumption of contaminated water and food, poor environmental hygiene, and under-developed sanitary systems [6,7]. Many among the Omani population, as with the family in this case report, have social relationships and bonds with people living in Pakistan, India, Yemen, and Tanzania. Thus, the ongoing war and conflicts in Yemen add further pressure and risk of the disease spreading to Oman.

The communicable disease manual of the Omani Ministry of Health defines a suspected case of cholera as a patient aged 5 years or more who develops severe dehydration, or who dies from acute watery diarrhoea, or who has a history of travel from a country with an ongoing outbreak of cholera and shows symptoms within 5 days of arrival in Oman [4].

In the case of the father described herein, the patient was successfully diagnosed as a suspected case of cholera upon his arrival at hospital. He presented with a severe manifestation of cholera, and he was deteriorating rapidly. The detailed history of the patient’s recent travel added to the high possibility of cholera, and, consequently, public health and infection control actions were immediately put in place, including isolation of the patient, full enteric precautions, and notification and involvement of the public health department. This step effectively prevented further possible infection transmission inside the hospital and at the patient’s home.

However, this was not the case at the primary health care venue where the patient was first seen. Even the severe presentation of cholera did not alert the treating physician, and no travel history was taken. Thus, the first primary health care encounter missed the diagnosis of a clear-cut suspected cholera case, and this was most likely the place where the daughter acquired the infection. The infection control role within the polyclinic was absent in this case, and the patient’s relatives were openly providing care to the father by cleaning his oral secretions and vomitus without being offered any precautions.

In terms of the daughter’s scenario, which demonstrated a mild to moderate presentation, the family had denied the existence of any other sick members during the first interview with the public health team. However, the primary health centre where the second patient sought medical advice for her persistent loose bowel movements and abdominal cramps also failed to uncover the cause of her problems, and another suspected cholera case was missed. The incubation period for the daughter can be estimated as being 2 days, while that of the father was hard to determine exactly. Generally, the cholera incubation period may be between several hours and 5 days (typically 1-2 days) [8]. It is also crucial to realise that the *V. cholerae* can be detected in the stool 1 day to 10 days after infection, which represents a period in which it may be shed into the environment; during this period, the majority of patients do not develop any symptoms [9].

These cases indicate 2 major areas for improvement in Oman: public health surveillance and infection control programmes. The main objective for 2030 in the Global Roadmap to End Cholera is early detection and quick response to contain outbreaks at the earliest stages [10]. This can, however, only be achieved by strengthening the integrated early warning surveillance systems, improving laboratory capacity, and utilising rapid diagnostic tests [10]. Screening contacts through the RDT did result in the detection of a second case of cholera despite the patient not admitting her symptoms on the first visit of the public health team. Different RDTs have been studied in a number of outbreak situations previously, and these studies have indicated some variations in sensitivity and specificity. For instance, Haiti’s experience showed high sensitivity and low specificity for both Artron and Crystal VC RDTs [11]. Significantly, the South Sudan outbreak indicated that the Crystal VC RDT, when used with a simple step of enrichment in alkaline peptone water, demonstrated performance similar to that of culture [12]. RDTs are thus fast, practical, and effective tests for large-scale epidemics where containment of the infection from the beginning is critical [13]. While stool culture is still the gold standard for diagnosing cholera, the time required to obtain results, combined with deficiencies in laboratory infrastructure, sample transportation, and staff training, can significantly delay detection and control actions [12]. The sensitivity of the culture can also be affected by prior consumption of antibiotics or sampling transportation [14].

In view of Oman’s current situation, RDTs would thus undoubtedly be of great value in terms of the early detection of suspected cases to prevent further secondary infections, and indeed, RDTs worked exceptionally well in both cases reported here. It is also important to highlight the pivotal role of community awareness. Previous studies in Bangladesh have showed that people’s knowledge and awareness of cholera is poor, while in Tanzania, the actual practices of people in terms of reducing cholera transmission were low [15,16]. The 2 patients reported here were aware neither of cholera endemicity in Pakistan nor the serious complications of the disease itself. Raising awareness and knowledge of cholera and good hygiene practices, especially for Omani travellers going abroad, is thus an important step that must be implemented by the public health authorities.

Based on the discussion above, it is clearly important to strengthen public health surveillance in primary health care to detect cholera cases early and prevent further secondary infections. This should be done in parallel with establishing and improving infection control programmes. These case studies also support the distribution of RDTs all over Oman to allow rapid screening and monitoring of contacts, along with increasing cholera awareness and knowledge of good hygiene practices, especially for travellers outside of the country.
